# Health-seeking behavior among hematuria patients in Jordan: the case of the Health Belief Model

**DOI:** 10.3389/fpubh.2026.1875282

**Published:** 2026-08-06

**Authors:** Mohammad Abufaraj, Jood Al-Adwan, Tamara Al-Azzeh, Yara Saddam, Abdalrhman Albassomi, Mohammad Al-Sabbagh, Safwan Abu Khalaf, Abdulrahman Yamin, Amr S. Khatatneh, Baha Aldeen Alshraideh, Hana Taha

**Affiliations:** 1 Division of Urology, Department of Special Surgery, Jordan University Hospital, The University of Jordan, Amman, Jordan; 2 Department of Urology, Medical University of Vienna, Vienna, Austria; 3 Department of Internal Medicine, The National Center for Diabetes Endocrinology and Genetics, Amman, Jordan; 4 Department of Emergency Medicine, Jordan University Hospital, The University of Jordan, Amman, Jordan; 5 Department of Pediatrics, Jordan University Hospital, The University of Jordan, Amman, Jordan; 6 Division of Neurosurgery, Department of Special Surgery, Jordan University Hospital, The University of Jordan, Amman, Jordan; 7 Department of Neurology, University of Kansas Medical Center, Kansas City, KS, United States; 8 Faculty of Medicine, Ibn Sina University for Medical Sciences, Amman, Jordan; 9 Department of Family and Community Medicine, School of Medicine, The University of Jordan, Amman, Jordan; 10 Department of Neurobiology, Care Sciences and Society, Karolinska Institutet, Solna, Sweden

**Keywords:** Health Belief Model, health-seeking behavior, hematuria, Jordan, urology

## Abstract

**Background/objectives:**

Despite broad application of the Health Belief Model (HBM) across a range of clinical contexts, its relevance to patients actively presenting with hematuria remains unexplored. Hematuria may stem from minor self-resolving causes or serious malignancies requiring early intervention. This study aimed to characterize health-seeking behavior in this population and to determine which clinical and belief-based factors predict whether patients pursue medical evaluation.

**Methods:**

We conducted a cross-sectional study on 150 patients presented with hematuria, enrolled by convenience sampling from a single tertiary-care urology center. Participants completed a self-administered Arabic questionnaire comprising the Champion Revised Health Belief Model Scale and a sociodemographic instrument. Binary logistic regression was used to identify independent predictors of care-seeking. Health-seeking behavior was analyzed in 125 of the 150 enrolled patients; 25 participants were excluded because hematuria was detected solely on urinalysis without personal symptom awareness.

**Results:**

Macroscopic hematuria accounted for 68% of cases and was more common among males (74.5%) and those older than 70 years (85%). Overall, 91% of participants sought or were actively seeking medical care. Among clinical and belief-based variables tested, only hematuria type reached marginal statistical significance: macroscopic presentation was associated with a 2.7-fold higher likelihood of care-seeking relative to microscopic (OR = 2.66, 95% CI 1.00–7.08, *p* = 0.049). This finding should be interpreted with caution given the marginal *p*-value and a confidence interval whose lower bound is at 1.0. No HBM construct—including perceived susceptibility, severity, benefits, barriers, confidence, or health motivation—independently predicted this outcome.

**Conclusion:**

When a symptom is as visually striking as gross hematuria, the decision to seek care appeared to be driven primarily by symptom visibility rather than belief-based constructs, though causal inferences cannot be drawn from this cross-sectional, single-center study. The model’s six constructs did not independently predict care-seeking once hematuria type was considered, suggesting limited additive utility in this context; however, replication in larger and more diverse samples is needed before broader conclusions about HBM applicability are drawn. Importantly, patients with microscopic hematuria—whose condition is no less clinically serious—were less likely to seek care, pointing to a gap that warrants direct attention in future public health planning.

## 1 Introduction

Hematuria is defined as the presence of red blood cells in the urine that encompasses two clinically distinct presentations. Macroscopic hematuria is visible to the patient and tends to prompt immediate concern; microscopic hematuria, by contrast, is often discovered incidentally during routine urinalysis in an otherwise asymptomatic individual (1). The differential diagnosis spans a wide spectrum, from simple conditions such as urinary tract infection to urological malignancies that carry significant mortality if diagnosed late. In clinical practice, macroscopic hematuria warrants urgent referral to a urologist, while microscopic hematuria can elicit the same urgency in certain risk factors such as significant smoking history and for men over the age of 60 years old (2).

What drives a patient with hematuria to seek care is a question the literature has largely left unanswered. This matters for a straightforward reason: delayed presentation is associated with more advanced disease at diagnosis, particularly for bladder and renal malignancies. The Health Belief Model (HBM), developed in the 1950s to explain why individuals do or do not adopt preventive health behaviors (3, 4)offers a useful lens through which to examine these motivations. The model posits six constructs: perceived susceptibility, perceived severity, perceived benefits, perceived barriers, cues to action, and self-efficacy (the last added in 1988). These constructs are theoretically independent, and each is thought to account for a portion of the variability seen in health-related behaviors (5).

A previous study in Jordan assessed HBM constructs in a urology context and found that perceived severity, perceived benefits, and perceived barriers were the strongest predictors of health-seeking behavior, with perceived seriousness also emerging as a key determinant of care-seeking delay (6, 7). A study from China focused on colorectal cancer screening compliance reached a broadly similar conclusion which showed that perceived severity was the dominant driver of colonoscopy uptake relative to the model’s other components (8).

While preventive screenings rely heavily on an individual’s abstract calculation of risk, symptom-driven healthcare utilization behaves under entirely different dynamics. Multi-country European studies demonstrate that the physical features of a symptom—particularly its visibility and perceived threat level—frequently override standard socio-cognitive timelines (9, 10).

This study focuses on a symptom rather than a screening examination and the inquiry is not about their willingness to undergo a preventive procedure, but rather whether they will respond to an active and potentially alarming symptom and what drives that response. To the best of our knowledge, no previous studies have applied the HBM to investigate the factors associated with health-seeking behavior in patients presenting with hematuria. Although the HBM was originally developed to explain preventive behaviors, its constructs—particularly perceived severity and perceived barriers—are conceptually applicable to responses to active symptoms; a patient’s appraisal of the seriousness of hematuria and the obstacles to obtaining care may plausibly determine whether and how quickly they present for evaluation. We hypothesized that higher perceived severity and lower perceived barriers would be independently associated with a greater likelihood of health-seeking behavior. Therefore, this study aimed to examine the attitudes and beliefs of hematuria patients and how these constructs predict their health-seeking behavior.

## 2 Methodology

### 2.1 Study setting

The study was conducted at Jordan University Hospital (JUH), a tertiary hospital and one of the principal academic centers in Amman. Jordan is a higher-middle-income country with a population of roughly 10 million and a healthcare landscape that mixes public, military, university, and private insurance schemes—a context that shapes both access to care and health-seeking patterns (11).

### 2.2 Study design and sampling

This was a cross-sectional study enrolling 150 patients attending the urology clinics at JUH between November 1st, 2024 and November 11th, 2025. Based on an anticipated care-seeking prevalence of 80%, a 95% confidence level, and a margin of error of 7%, the minimum required sample size was estimated to be 126 participants. The final sample of 150 participants exceeded this requirement. Participants were selected by convenience sampling and were eligible if they aged 17–90, presenting with undiagnosed hematuria, mentally competent, and able to comprehend and communicate in Arabic. Patients with a prior diagnosis of bladder, prostate, renal, or colorectal cancer; females who were pregnant or menstruating at the time of the visit; individuals working in the medical field; those who had undergone recent urinary tract procedures; and those with a history of recent pelvic or abdominal trauma or road traffic accident were excluded. These exclusions were intended to ensure that the presenting symptom was undiagnosed and that participants were responding to hematuria as a novel clinical concern rather than a recurrent or professionally demystified one.

### 2.3 Study participants

The sociodemographic and clinical characteristics of participants are summarized in Table 1, stratified by hematuria presentation (microscopic or gross/macroscopic). The sample was predominantly male (70.7%) and married (78.7%), consistent with the demographic profile of urology clinic attendees. Macroscopic hematuria accounted for 68% of cases overall; it was more prevalent in males (74.5% vs. 52.3% in females, *p* < 0.01) and was most common in those older than 70 years (85%). In terms of smoking history, 39.3% of the total sample identified as active smokers, with a higher percentage observed in the macroscopic group compared to the microscopic group (43.1% vs. 31.2%), though this difference was not statistically significant (*p* = 0.226). Patients under 40, by contrast, had the highest proportion of microscopic hematuria (42.5%). The distribution of hematuria type did not differ significantly by income (*p* = 0.5), educational level (*p* = 0.6), or marital status (*p* = 0.3).

**Table 1:** Sociodemographic characteristics of 150 participants according to hematuria type.

Categories	Total *n* (%)	Macroscopic *n* (%)	Microscopic *n* (%)	*p* value
Overall	150 (100)	102 (68)	48 (32)	
Age (years)				0.2
<40	40 (26.7)	23 (57.5)	17 (42.5)	
41–50	30 (20.0)	21 (70.0)	9 (30.0)	
51–60	36 (24.0)	23 (63.9)	13 (36.1)	
61–70	24 (16.0)	18 (75.0)	6 (25.0)	
>70	20 (13.3)	17 (85.0)	3 (15.0)	
Gender				<0.01
Male	106 (70.7)	79 (74.5)	27 (25.5)	
Female	44 (29.3)	23 (52.3)	21 (47.7)	
Smoking status				0.226
Non-smoker	91 (60.7)	58 (56.9)	33 (68.8)	
Smoker	59 (39.3)	44 (43.1)	15 (31.2)	
Income (JOD)^*^				0.5
<700	80 (53.3)	55 (68.8)	25 (31.3)	
701–1,500	57 (38.0)	40 (70.2)	17 (29.8)	
>1,501	13 (8.7)	7 (53.9)	6 (46.2)	
Education				0.6
Less than secondary	23 (15.3)	19 (82.6)	4 (17.4)	
Secondary	23 (15.3)	16 (69.6)	7 (30.4)	
Diploma	25 (16.7)	15 (60.0)	10 (40.0)	
University	42 (28.0)	27 (64.3)	15 (35.7)	
Higher education	26 (17.3)	18 (69.2)	8 (30.8)	
Started university	11 (7.3)	7 (63.6)	4 (36.4)	
Marital status				0.3
Single	24 (16.0)	14 (58.3)	10 (41.7)	
Married	118 (78.7)	84 (71.2)	34 (28.8)	
Divorced	8 (5.3)	4 (50.0)	4 (50.0)	

^*^Monthly household income in JOD = Jordanian Dinar; 1 JOD ≈ 1.41 USD. ^†^‘Started university’ refers to participants who enrolled in undergraduate study but did not complete a degree; this category does not include currently enrolled students.

### 2.4 Study instrument development and validation

Data were collected using two instruments. The first was the Champion Revised Health Belief Model Scale (CHBMS), originally translated into Arabic and adapted for use in Jordan (12) and was subsequently modified by our team to reflect the clinical context of hematuria (13). The latter was a researcher-developed sociodemographic questionnaire. All participants provided written and verbal informed consent and completed both instruments via personal interview using Google Forms at the urology clinic; completion took approximately 7 min.

The modified CHBMS comprises 42 items across six subscales: perceived susceptibility (5 items, range 5–25), perceived seriousness (7 items; range 7–35), perceived benefits (6 items; range 6–30), perceived barriers (6 items, range 6–30), perceived confidence (11 items, range 11–55), and health motivation (7 items, range 7–35). Responses were recorded on a five-point Likert scale. For all subscales except barriers, higher scores indicate more favorable health attitudes; on the barrier’s subscale, a higher score reflects greater perceived obstacles to care. An additional response option “I do not know about that test” was introduced in the confidence subscale to capture lack of familiarity with home dipstick urinalysis testing rather than opposition to its use. This response was scored as 1 (equivalent to “strongly disagree”/no confidence), reflecting the premise that unfamiliarity with the test represents an absence of self-efficacy rather than a neutral position. This coding decision was made a prior during instrument adaptation.

The questionnaire was piloted in 30 patients not included in the main study. Internal consistency was acceptable across all subscales: Cronbach’s alpha coefficients were 0.78 for perceived susceptibility, 0.86 for perceived seriousness, 0.91 for perceived benefits, 0.61 for perceived barriers, 0.78 for perceived confidence, and 0.72 for health motivation. The total scale alpha was 0.82. It should be noted that the perceived barriers subscale yielded a Cronbach’s alpha of 0.61, indicating modest internal consistency. As lower reliability can reduce the ability to detect true associations, the null findings for this construct should be interpreted with caution.

The sociodemographic questionnaire covered 30 items addressing age, gender, education, marital status, house hold income, insurance status, smoking habits, hematuria type, family and personal history of hematuria, anticoagulant use, and prior knowledge of hematuria and its potential causes.

### 2.5 Data analysis

Statistical analysis was carried out in STATA (Release 19SE StataCorp LLC, College Station, TX). The significance threshold was set at *p* < 0.05 throughout. Continuous variables were reported as means with standard deviations; categorical variables were expressed as frequencies and percentages. Chi-square tests were used to examine associations between categorical variables and hematuria type. To identify factors associated with care-seeking behavior, univariable logistic regression analyses were initially performed for demographic variables (including age, sex, educational level, and other relevant clinical characteristics) as well as Health Belief Model (HBM) constructs. Demographic and clinical variables were evaluated as potential confounders. However, none demonstrated a statistically significant association with care-seeking behavior in univariable analyses. Given the limited sample size and the small number of participants who did not seek medical care, inclusion of a large number of predictors in the multivariable model was considered likely to result in model overfitting. Therefore, only variables deemed clinically relevant to the study objective, including hematuria type and HBM constructs, were retained in the final multivariable logistic regression model. Adjusted odds ratios (ORs) with 95% confidence intervals (CIs) were calculated. Multicollinearity among predictors was evaluated using variance inflation factors (VIFs), with values greater than 5 considered indicative of potentially problematic collinearity. Missing data were handled using complete-case analysis. Participants with missing values for variables included in a given analysis were excluded from that specific analysis, resulting in variable sample sizes across statistical tests.

## 3 Results

**Figure 1:**
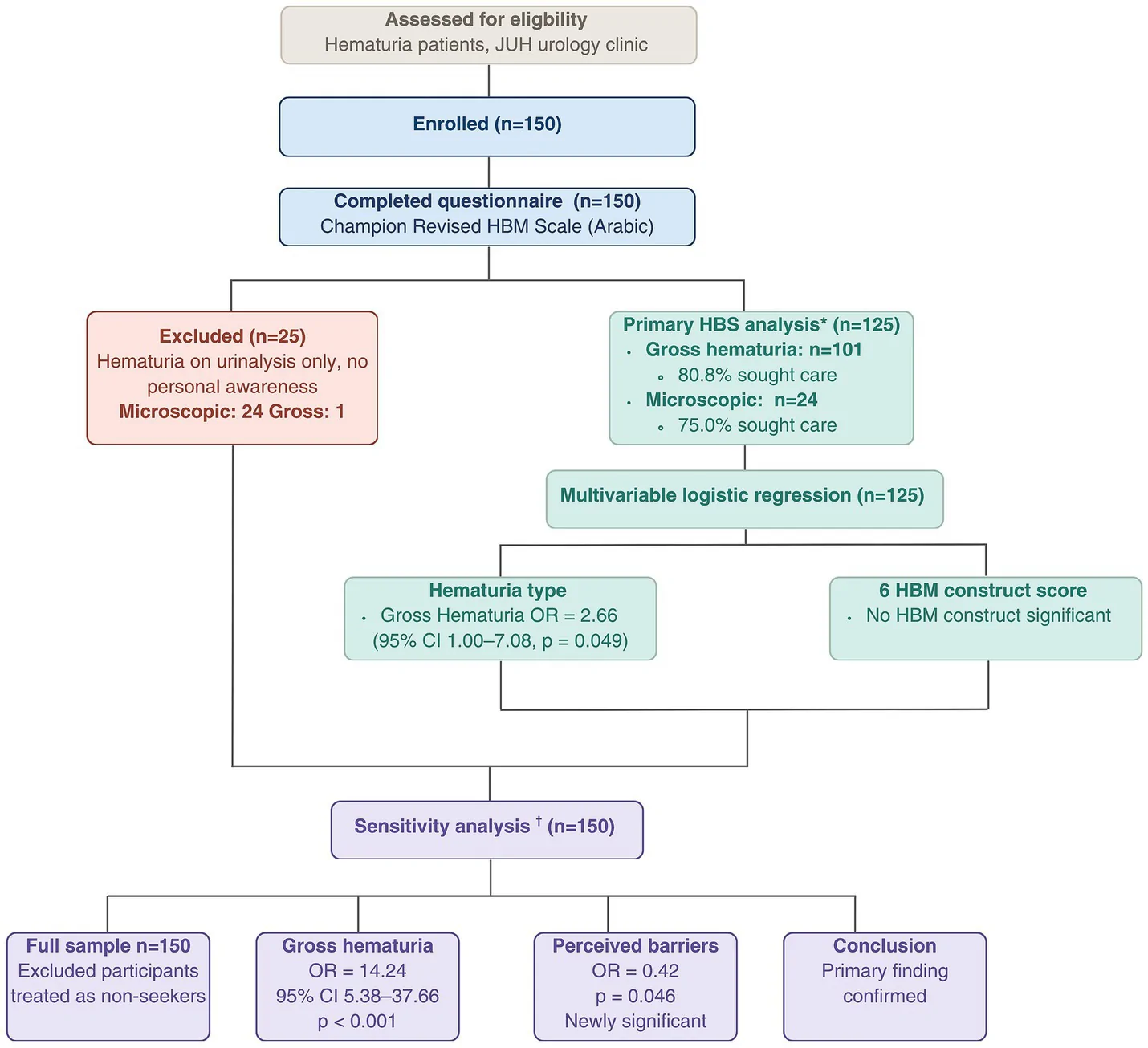
Study population selection and analytical framework. HSB, Health-Seeking Behavior; HBM, Health Belief Model; JUH, Jordan University Hospital; OR, odds ratio; Cl, confidence interval. ^*^The primary analysis (*n* = 125) excluded 25 participants whose hematuria was detected solely on urinalysis without personal symptom awareness (24 microscopic, 1 gross); these individuals could not meaningfully respond to the primary HSB outcome (whether they visited a doctor after observing hematuria). ^†^The sensitivity analysis (*n* = 150) reclassified the 25 excluded participants as non-seekers to assess the robustness of the primary finding. Hematuria type remained the dominant predictor, with the effect size strengthened (OR = 14.24 vs. 2.66 in the primary analysis). Perceived barriers reached statistical significance in the sensitivity model only (OR = 0.42, *p* = 0.046), suggesting the primary model may have been underpowered to detect this association.

A participant flow diagram illustrating enrollment, exclusion, and the analytical samples used in the primary and sensitivity analyses is presented in Figure 1.

Table 1 presents the sociodemographic profile of the 150 participants by hematuria type. Macroscopic hematuria predominated (*n* = 102, 68%), and its distribution was skewed toward older males: 85% of patients over 70 had macroscopic hematuria, and males overall accounted for 74.5% of macroscopic cases compared to 52.3% of females. Age, income, education, and marital status did not differ significantly between the two hematuria groups. Gender, however, was a significant determinant, with males demonstrating a significantly higher prevalence of macroscopic hematuria (*p* = 0.008).

Table 2 summarizes the health-seeking behavior of 125 patients according to relevant health-related factors. Because health-seeking behavior was defined as a response to the patient’s own recognition of hematuria, the 25 participants who reported no personal awareness of the symptom—the majority of whom had microscopic hematuria detected solely on urinalysis—were excluded from this analysis, yielding an analytic sample of 125 patients. Health-seeking was high across the board: 91% of patients had sought or were seeking medical care. Among those with macroscopic hematuria, 93.9% sought care, compared to 86.0% of those with microscopic hematuria—a difference that, while clinically plausible, did not reach statistical significance (*p* = 0.1). Family history of hematuria (*p* = 0.2), personal history of hematuria (*p* = 0.9), health insurance status (*p* = 0.1), prior knowledge of hematuria or its causes and anticoagulant use (*p* = 0.4 and *p* = 0.5, respectively), were similarly non-significant predictors. No missing data were identified in the primary outcome variable or the HBM subscale scores. Where individual questionnaire items were missing, the case was excluded from the relevant subscale calculation.

**Table 2:** Health factors of 125 patients in terms of health-seeking behavior (HSB).

Variable	Total (%)	HSB yes *n* (%)	HSB no *n* (%)	*p* value
Overall health-seeking behavior	125 (100)	114 (91)	11 (9)	
Type of hematuria				
Macroscopic	82 (65.6)	77 (93.9)	5 (6.1)	0.1
Microscopic	43 (34.4)	37 (86.0)	6 (14.0)	
Family history				0.2
Positive	13 (10.4)	11 (84.6)	2 (15.4)	
Negative	87 (69.6)	82 (94.3)	5 (5.7)	
Unknown	25 (20.0)	21 (84.0)	4 (16.0)	
Personal history of hematuria				0.9
Yes	68 (54.4)	62 (91.2)	6 (8.8)	
No	56 (44.8)	51 (91.1)	5 (8.9)	
Unknown	1 (0.8)	1 (100.0)	0 (0.0)	
Health insurance				0.1
Insured	116 (92.8)	107 (92.2)	9 (7.8)	
Uninsured	9 (7.2)	7 (77.8)	2 (22.2)	
Previous knowledge about hematuria				0.4
Yes	59 (47.2)	52 (88.1)	7 (11.9)	
No	66 (52.8)	62 (93.9)	4 (6.1)	
Use of blood thinners				0.5
Yes	45 (36.0)	40 (88.9)	5 (11.1)	
No	80 (64.0)	74 (92.5)	6 (7.5)	

Twenty-five participants (24 microscopic, 1 macroscopic) were excluded as they had not personally observed blood in their urine; their hematuria was detected incidentally on urinalysis. Health-seeking behavior was therefore analyzed in the remaining 125 patients. The relatively high proportion of unknown family history (*n* = 25, 20%) likely reflects poor recall of family medical history rather than a true absence of family history; this should be borne in mind when interpreting the family history subgroup results.

Table 3 shows the multivariate binary regression results. Of all the variables, Hematuria type was the only variable that reached statistical significance, with patients presenting with macroscopic hematuria being significantly more likely to seek medical care than those with microscopic hematuria (OR = 2.66, 95% CI 1.00–7.08, *p* = 0.049). This result should be interpreted with caution, as the *p*-value is only marginally significant and the lower bound of the confidence interval approaches 1.0, indicating uncertainty around the magnitude of the effect. None of the HBM constructs—perceived susceptibility (OR = 0.89, *p* = 0.3), perceived seriousness (OR = 0.94, *p* = 0.3), perceived benefits (OR = 0.94, *p* = 0.6), perceived barriers (OR = 0.88, *p* = 0.1), perceived confidence (OR = 1.01, *p* = 0.9), and health motivation (OR = 0.91, *p* = 0.3)—reached statistical significance. No substantial multicollinearity was observed among predictors, with all variance inflation factors below 5. The model explained a modest proportion of the variability in care-seeking behavior (McFadden pseudo-*R*^2^ = 0.08).

**Table 3:** Multivariate binary regression analyses of HBM constructs affecting health-seeking behavior in 125 participants.

Subgroups	Adjusted OR	Regression coefficient	Standard error	*p* value	95% confidence interval
Type of hematuria (macroscopic vs. microscopic)	2.66	0.99	0.50	0.049	1.00–7.08
Perceived susceptibility	0.89	−0.12	0.11	0.277	0.72–1.10
Perceived seriousness	0.94	−0.06	0.06	0.295	0.84–1.05
Perceived benefits	0.94	−0.06	0.12	0.626	0.74–1.20
Perceived barriers	0.88	−0.12	0.08	0.134	0.75–1.04
Perceived confidence	1.01	0.01	0.05	0.857	0.91–1.12
Health motivation	0.91	−0.10	0.10	0.313	0.75–1.10

OR, odds ratio; CI, confidence interval; SE, standard error. Standard errors are reported alongside confidence intervals to facilitate post-hoc power calculations and comparison with future studies; where CIs are sufficient for clinical interpretation, SEs may be omitted in the final published version at editorial discretion.

## 4 Discussion

In this sample of Jordanian patients presenting with hematuria, a substantial majority (91%) demonstrated health-seeking behavior, reflecting a generally favorable disposition toward seeking medical evaluation. The central finding of this study is straightforward, though its interpretation is more complex. Among all variables examined—sociodemographic characteristics, clinical history, insurance coverage, prior knowledge, and six HBM constructs—only hematuria type significantly predicted whether patients sought medical care. Patients with visible hematuria were 2.7 times more likely to present for evaluation than those whose hematuria was detectable only on urinalysis. The HBM constructs, taken individually, did not add explanatory power. These observations are consistent with previous reports demonstrating that the HBM’s applicability may vary depending on the clinical context and the population under study (14).

We interpret this finding as reflecting the overriding salience of macroscopic hematuria as a symptom. When blood in the urine is visible, the decision to seek care may be less a product of deliberative belief-weighting—the cognitive process the HBM is designed to capture—and more an almost reflexive response to a frightening and unmistakable sign. This interpretation is at least consistent with the model’s own assumptions: the HBM was conceived to explain variation in health behavior, and where a symptom is sufficiently alarming to drive near-universal care-seeking (93.9% in our macroscopic group), there may simply be insufficient variation to detect.

This overriding influence of symptom visibility aligns with broader international data on urological malignancies. Prior research on patient care-seeking journeys indicates that high symptom salience—such as visible blood—frequently prompts an immediate, almost reflexive transition from symptom recognition to care utilization, effectively compressing the timeline for deliberative cognitive processes (15). Consequently, when contrasted with cohorts presenting with vague or non-visible urological complaints where psychological and structural barriers significantly dictate delays (16), the sheer visual alarm of gross hematuria establishes a statistical ceiling effect, minimizing the measurable variance that HBM constructs are designed to catch. Therefore, this interpretation should be viewed as exploratory rather than conclusive; future studies recruiting beyond specialist urology clinics would be needed to confirm it, particularly given the risk of Berkson’s bias inherent to clinic-based sampling, which likely inflates observed care-seeking rates and narrows the range of HBM construct scores.

The demographic profile of our sample bears resemblance to that reported in earlier studies. The predominance of married, male participants aligns with earlier findings on myocardial infarction care-seeking behavior in Jordan, where a similarly composed sample was observed (89.2% married, 68.7% male) (7). Health insurance coverage in our cohort was high (92.8%), with governmental insurance being the most common type, closely matching the 89.4% insured rate previously reported, of whom 56% had governmental coverage (6). However, our data differ from international reports in terms of educational attainment and income, both of which were lower in our sample compared with studies conducted in higher-income settings (15). This discrepancy likely reflects the socioeconomic context of Jordan rather than characteristics specific to patients with hematuria.

The relatively high proportion of macroscopic hematuria in our sample (68%) warrants some contextual consideration. Population-based epidemiological studies have reported lower rates, with gross hematuria accounting for only 35.7% of cases, while microscopic hematuria constitutes the majority at 62% (17). Additionally, the clinical prevalence of microscopic hematuria in the general population has been estimated at 4–5%, far exceeding rates of microscopic presentation (1). This discrepancy likely reflects the clinical setting rather than a true epidemiological pattern. Patients who notice visible blood in their urine are considerably more likely to seek urgent care, whereas those with microscopic hematuria—often detected incidentally during routine investigations—may never present at all (1). This self-selection dynamic may have biased our sample toward more symptomatic cases. Furthermore, in Jordan, access to urology specialty clinics differs markedly from primary care settings, which may further concentrate symptomatic cases.

Health Belief Model constructs have been shown to predict care-seeking behavior in other urological contexts (18). However, those studies were conducted in settings with more limited healthcare access, making direct comparisons with our tertiary clinic in Jordan challenging. In a more comparable context, perceived severity, benefits, and barriers were identified as the most significant predictors of prostate cancer screening behavior (6). The absence of these constructs in our findings is likely related to the nature of the presenting symptom itself: patients with hematuria—particularly macroscopic hematuria—are not making decisions based on probabilistic risk assessments but rather responding to an overt and alarming symptom. The visual nature of hematuria likely dominates decision-making, leaving limited scope for variation based on belief-driven factors. One finding worth examining more closely is the non-significant role of family history. We had anticipated that a positive family history of hematuria might heighten perceived susceptibility and, through that pathway, increase care-seeking. In practice, roughly 70% of our patients reported a family history of hematuria, yet this did not translate into meaningfully higher care-seeking rates. This may suggest that family history is too common in this population to function as a discriminating risk signal, or that patients do not readily link family history to their own symptoms in a way that activates health-seeking behavior.

Although external variables such as health insurance coverage and prior knowledge of hematuria were not significant predictors of health-seeking behavior in our study, this should not diminish their relevance to health policy. Across all age groups, most patients with hematuria were not adequately evaluated or referred for further investigation, highlighting persistent gaps in care delivery that extend beyond the patient’s decision to seek care (19).

Our results should be interpreted considering several limitations. First, the study utilized a convenience sampling method and recruited participants exclusively from the urology clinics at a tertiary medical center, which may limit the generalizability of findings to the broader population. Second, the cross-sectional design precludes the establishment of causal relationships between the study variables. Third, the relatively small sample size, while sufficient for the primary analysis, may have reduced statistical power to detect modest associations between HBM constructs and health-seeking behavior. Additionally, a subgroup analysis focusing specifically on patients with microscopic hematruria was not performed, which may have obscured belief-based predictors relevant to this clinically important but less symptomatic group. Finally, recall bias is also a concern, given that participants were asked to report beliefs that may have been formed or revised in the context of an active and potentially distressing symptom. Furthermore, the recruitment of participants exclusively from a tertiary-care urology clinic substantially restricts the generalizability of findings: individuals who never sought care are inherently absent from this sample, which may have contributed to the inflated overall rate of health-seeking behavior (91%) observed. This design feature limits the ability to investigate true determinants of initial care-seeking in the broader population. Regarding the 25 excluded participants, 24 had microscopic hematuria and 1 had macroscopic hematuria; their characteristics are described in Supplementary Table S1. A sensitivity analysis was conducted including all 150 participants, with the 25 incidentally detected cases classified as non–health seekers; results are presented in Supplementary Table S2. Notably, hematuria type did not significantly predict HSB-1 in the sensitivity analysis (*p* = 0.831), suggesting that the marginally significant OR observed in the primary analysis (OR = 2.66, *p* = 0.049) may in part reflect the composition of the excluded group rather than a robust association. No statistically significant associations were identified between HBM constructs and HSB. However, the study was powered to estimate the prevalence of HSB rather than to detect associations in multivariable logistic regression. Therefore, insufficient statistical power to detect modest associations cannot be excluded.

The possibility of Type II error also deserves consideration. The high prevalence of care-seeking behavior (91%) resulted in a small number of non-care-seeking participants, limiting statistical power for multivariable analyses. Therefore, the absence of significant associations between Health Belief Model constructs and care-seeking behavior should be interpreted cautiously and warrants confirmation in larger studies.

Cultural and healthcare system factors specific to Jordan may also have shaped the observed patterns. Jordan’s mixed public-private healthcare landscape, combined with high insurance coverage in this sample (92.8%), may have reduced structural barriers to care-seeking and thereby compressed variation in health-seeking behavior. Additionally, cultural norms around illness, gender roles, and family involvement in health decisions—which are prominent in the Jordanian context—were not formally captured in this study and may confound the relationship between HBM constructs and care-seeking behavior. Future research should incorporate culturally adapted measures and explore the role of social and familial influences on health behavior in this population.

The lower rate of care-seeking among patients with microscopic hematuria has direct public health implications. Because microscopic hematuria is invisible to the patient and is often detected only incidentally, many individuals with clinically significant underlying pathology may never present for evaluation. Targeted educational interventions—particularly among high-risk populations such as older males and smokers—are warranted to improve awareness that any form of hematuria, visible or not, merits prompt urological assessment. Community-based screening programs and primary care engagement may offer additional entry points to reach individuals who would not otherwise attend a specialist clinic.

## 5 Conclusions and recommendations

To our knowledge, this represents the first study to examine health-seeking behavior in patients with hematuria through the framework of the Health Belief Model. The central finding is, in many ways, a straightforward one: the visibility of a symptom appears to weigh heavily on whether a patient decides to seek care or not. Macroscopic hematuria emerged as by far the strongest predictor of care-seeking, while none of the six HBM constructs demonstrated an independent contribution to explaining this behavior. This should not be read as evidence that health beliefs bear no relevance to hematuria management. Rather, it may suggest that the predictive utility of the HBM is diminished when a symptom is sufficiently overt that deliberative belief processes become secondary to the immediate perceptual experience.

From a public health standpoint, these findings highlight a real gap: patients with microscopic hematuria, whose condition is less visually alarming but no less clinically important, were less likely to seek care. Raising awareness of hematuria as a symptom that warrants evaluation regardless of whether its visible appearance, promoting routine health screenings, particularly among older males and reducing structural barriers to diagnostic access are all reasonable targets for future intervention. Future studies should use larger prospective samples to better assess HBM constructs. Multicenter designs that incorporate community-based recruitment would be particularly valuable, as they would enable capture of individuals who do not seek care and thereby address the fundamental sampling limitation of the present study. Prospective designs, in which beliefs are assessed prior to the care-seeking decision, would allow stronger causal inference. Evaluation of complementary behavioral frameworks—such as the Theory of Planned Behavior or the Extended Parallel Process Model—may also yield additional insight into the determinants of health-seeking behavior in symptomatic patients. Frameworks that incorporate symptom salience as an explicit variable may more accurately capture care-seeking behavior in patients with active clinical signs.

## Data Availability

The raw data supporting the conclusions of this article will be made available by the authors, without undue reservation.
