# Supplementary material for: Health-seeking behavior among hematuria patients in Jordan: the case of the Health Belief Model
Source: Front Public Health. 2026 Aug 6;14:1875282. doi: 10.3389/fpubh.2026.1875282 (PMC13491194; doi:10.3389/fpubh.2026.1875282)
Supplement: Supplementary file 1 [file Data_Sheet_1.PDF]

STROBE Checklist — Cross-Sectional Studies

Health-Seeking Behavior in Hematuria Patients: An Application of the Health Belief Model

Manuscript ID: 1875282

This checklist follows the STROBE Statement for cross-sectional studies (von Elm E et al., PLoS Med 2007;4:e296). Status: ✓ = addressed | ~ = partially addressed | ✗ = not addressed.

No.	Section/Topic	Recommendation	✓/~/✗	Location in manuscript
1	Title & Abstract	Indicate study design in title or abstract; provide informative summary of what was done and found.	✓	Title states 'cross-sectional study'. Structured abstract present.
2	Introduction Background	Explain the scientific background and rationale for the investigation.	✓	Introduction §1–3: hematuria epidemiology, HBM overview, rationale added in revision.
3	Introduction Objectives	State specific objectives, including any pre-specified hypotheses.	✓	Introduction final §: aim stated; hypothesis statement added in revision.
4	Methods Study design	Present key elements of study design early in the paper.	✓	Section 2.2: 'cross-sectional study'; convenience sample; single tertiary-care center.
5	Methods Setting	Describe the setting, locations, and relevant dates including periods of recruitment.	✓	Section 2.2: Jordan University Hospital, Amman, Jordan. Recruitment: November 1, 2024 – November 11, 2025 (confirmed accurate).
6	Methods Participants	Give eligibility criteria, sources and methods of selection of participants.	✓	Section 2.2: age 17–90, undiagnosed hematuria, mentally competent. Exclusion criteria stated.
7	Methods Variables	Clearly define all outcomes, exposures, predictors, confounders, and effect modifiers.	✓	Sections 2.3–2.4: HSB (outcome); hematuria type (predictor); 6 HBM subscales; sociodemographics.
8	Methods Data sources	For each variable, describe data sources and methods of assessment.	✓	Section 2.4: Champion Revised HBM Scale; Arabic translation; pilot testing n=30; Cronbach's α reported.
9	Methods Bias	Describe any efforts to address potential sources of bias.	~	Convenience sampling acknowledged. Berkson's bias named in Discussion (added in revision). No prospective design-stage bias mitigation — stated as limitation.
10	Methods Study size	Explain how the study size was arrived at.	✓	Section 2.2: sample size calculation — 80% prevalence, 95% CI, 7% margin → n≥126; 150 enrolled.
11	Methods Quantitative variables	Explain how quantitative variables were handled in the analyses.	✓	Section 2.4: HBM subscale total scores used as continuous variables in regression.
12 a	Methods Statistical methods	Describe all statistical methods, including those used to control for confounding.	✓	Section 2.5 (revised): univariable screening; multivariable logistic regression; STATA 19SE.
12 b	Methods Subgroup / sensitivity	Describe any methods used to examine subgroups and interactions.	✓	Sensitivity analysis (n=150) conducted and reported in revision.
12 c	Methods Missing data	Explain how missing data were addressed.	✓	Section 2.5: complete-case analysis; item-level exclusion for subscales stated.

No.	Section/Topic	Recommendation	✓/~/✗	Location in manuscript
12 d	Methods Sampling strategy	If applicable, describe analytical methods taking account of sampling strategy.	✓	Convenience sampling acknowledged; implications discussed in Limitations.
13 a	Results Participants	Report numbers at each stage; give reasons for non-participation. Consider flow diagram.	~	Table 2 footnote: 25 excluded (24 microscopic, 1 gross), reason stated. CONSORT flow diagram submitted as Supplementary File 1. Refusal data not recorded during recruitment — stated as limitation.
13 b	Results Participants	Give characteristics of study participants.	✓	Table 1: full sociodemographic and clinical characteristics by hematuria type.
14	Results Descriptive data	Indicate number of participants with missing data for each variable.	✓	Section 2.5 & Results: no missing data in primary outcome; item-level handling stated.
15	Results Outcome data	Report numbers of outcome events or summary measures.	✓	Table 2: HSB rates by clinical and demographic subgroup (n=125).
16 a	Results Main results	Give unadjusted and adjusted estimates and their precision.	✓	Table 3: adjusted OR, β, SE, 95% CI, p for all predictors. Univariable in Table 2.
16 b	Results Category boundaries	Report category boundaries when continuous variables were categorized.	✓	Age by decade; income by JOD ranges — stated in Table 1.
16 c	Results Absolute risk	If relevant, translate relative risk into absolute risk.	~	Cross-sectional design; absolute risk not applicable. McFadden R^2 reported as model-level effect size.
17	Results Other analyses	Report sensitivity analyses and subgroup analyses.	✓	Sensitivity analysis (n=150, 25 treated as non-seekers) added in revision; results reported.
18	Discussion Key results	Summarise key results with reference to study objectives.	✓	Discussion §1: key findings summarized in relation to stated objectives and hypotheses.
19	Discussion Limitations	Discuss limitations considering sources of bias; discuss direction and magnitude.	✓	Limitations: Berkson's bias (direction stated), ceiling effect, cross-sectional design, single-center, Type II error, exclusion bias, Cronbach's α, recall bias.
20	Discussion Interpretation	Give a cautious overall interpretation of results.	✓	Discussion: HBM utility framed cautiously; marginal OR caveat added; null findings attributed to possible underpowering.
21	Discussion Generalisability	Discuss the generalisability of the study results.	✓	Limitations: single-center convenience sample; Jordan-specific context discussed; community-based replication recommended.
22	Other Funding	Give the source of funding and the role of the funders.	✓	Funding section states: 'This research received no specific funding from public, commercial, or not-for-profit agencies.'

Summary: *24 of 27 items fully addressed (✓) | 3 items partially addressed (~): design-stage bias mitigation, refusal data not recorded, absolute risk not applicable to cross-sectional design.*

Reference: *von Elm E, Altman DG, Egger M, et al. The Strengthening the Reporting of Observational Studies in Epidemiology (STROBE) statement: guidelines for reporting observational studies. PLoS Med. 2007;4(10):e296.*
