# Supplementary material for: Health-seeking behavior among hematuria patients in Jordan: the case of the Health Belief Model
Source: Front Public Health. 2026 Aug 6;14:1875282. doi: 10.3389/fpubh.2026.1875282 (PMC13491194; doi:10.3389/fpubh.2026.1875282)
Supplement: Supplementary file 2 [file Table_1.docx]

**Supplementary Table 1. Baseline Characteristics of the 25 Participants Excluded from the Primary Health-Seeking Behavior Analysis (HSB-1)**

| **Variable** | **Excluded participants (N = 25), n (%)** |
| --- | --- |
| **Type of Hematuria** |  |
| Macroscopic | 1 (4.0) |
| Microscopic | 24 (96.0) |
| **Family History** |  |
| Positive | 9 (36.0) |
| Negative | 12 (48.0) |
| Unknown | 4 (16.0) |
| **Personal History of Hematuria** |  |
| Yes | 17 (68.0) |
| No | 8 (32.0) |
| **Health Insurance** |  |
| Insured | 25 (100.0) |
| Uninsured | 0 (0.0) |
| **Previous Knowledge About Hematuria** |  |
| Yes | 13 (52.0) |
| No | 12 (48.0) |
| **Use of Blood Thinners** |  |
| Yes | 2 (8.0) |
| No | 23 (92.0) |
