# Supplementary material for: Health-seeking behavior among hematuria patients in Jordan: the case of the Health Belief Model
Source: Front Public Health. 2026 Aug 6;14:1875282. doi: 10.3389/fpubh.2026.1875282 (PMC13491194; doi:10.3389/fpubh.2026.1875282)
Supplement: Supplementary file 3 [file Table_2.docx]

## Supplementary Table 2. Sensitivity Analysis: Health Factors Among All 150 Participants, with the 25 Incidentally Detected Cases Classified as Non–Health Seekers

| **Variable** | **Total (%)** | **HSB Yes n (%)** | **HSB No n (%)** | **P value** |
| --- | --- | --- | --- | --- |
| Overall | 150 (100) | 114 (76.0) | 36 (24.0) |  |
| **Hematuria Type** |  |  |  | 0.831 |
| Macroscopic | 102 (68.0) | 77 (75.5) | 25 (24.5) |  |
| Microscopic | 48 (32.0) | 37 (77.1) | 11 (22.9) |  |
| **Family History** |  |  |  | 0.004 |
| Positive | 22 (14.7) | 11 (50.0) | 11 (50.0) |  |
| Negative | 99 (66.0) | 82 (82.8) | 17 (17.2) |  |
| Unknown | 29 (19.3) | 21 (72.4) | 8 (27.6) |  |
| **Personal History** |  |  |  | 0.541 |
| Yes | 85 (56.7) | 62 (72.9) | 23 (27.1) |  |
| No | 64 (42.7) | 51 (79.7) | 13 (20.3) |  |
| **Health Insurance** |  |  |  | 0.898 |
| Insured | 141 (94.0) | 107 (75.9) | 34 (24.1) |  |
| Uninsured | 9 (6.0) | 7 (77.8) | 2 (22.2) |  |
| **Knowledge of Causes** |  |  |  | 0.009 |
| Yes | 70 (46.7) | 60 (85.7) | 10 (14.3) |  |
| No | 80 (53.3) | 54 (67.5) | 26 (32.5) |  |
| **Use of Blood Thinners** |  |  |  | 0.078 |
| Yes | 47 (31.3) | 40 (85.1) | 7 (14.9) |  |
| No | 103 (68.7) | 74 (71.8) | 29 (28.2) |  |
